# Efficacy and safety of percutaneous transforaminal endoscopic surgery (PTES) compared with MIS-TLIF for surgical treatment of lumbar degenerative disease in elderly patients: A retrospective cohort study

**DOI:** 10.3389/fsurg.2022.1083953

**Published:** 2023-04-17

**Authors:** Tianle Ma, Tianyao Zhou, Yutong Gu, Liang Zhang, Wu Che, Yichao Wang

**Affiliations:** ^1^ Department of Orthopedic Surgery, Zhongshan Hospital Fudan University, Shanghai, China; ^2^ Shanghai Southwest Spine Surgery Center, Shanghai, China

**Keywords:** lumbar degenerative disease, elderly patient, transforaminal, endoscopic surgery, transforaminal lumbar interbody fusion, minimally invasive spine surgery

## Abstract

**Objectives:**

To evaluate the efficacy and safety of PTES for surgical treatment of lumbar degenerative disease (LDD) including lumbar disc herniation, lateral recess stenosis, intervertebral foraminal stenosis and central spinal canal stenosis in elderly patients compared with MIS-TLIF.

**Methods:**

From November 2016 to December 2018, 84 elderly patients (>70 years old) of single-level LDD with neurologic symptoms underwent the surgical treatment. 45 patients were treated using PTES under local anesthesia in group 1 and 39 patients treated using MIS-TLIF in group 2. Preoperative, postoperative back and leg pain were evaluated using Visual analog scale (VAS) and the results were determined with Oswestry disability index (ODI) at 2-year follow-up. All complications were recorded.

**Results:**

PTES group shows significantly less operation time (55.6 ± 9.7 min vs. 97.2 ± 14.3 min, *P* < 0.001), less blood loss [11(2–32) ml vs. 70(35–300) ml, *P* < 0.001], shorter incision length (8.4 ± 1.4 mm vs. 40.6 ± 2.7 mm, *P* < 0.001), less fluoroscopy frequency [5(5–10) times vs. 7(6–11) times, *P* < 0.001] and shorter hospital stay[3(2–4) days vs. 7(5–18) days, *P* < 0.001] than MIS-TLIF group does. Although there was no statistical difference of leg VAS scores between two groups, back VAS scores in PTES group were significantly lower than those in MIS-TLIF group during follow-ups after surgery (*P* < 0.001). ODI of PTES group was also significantly lower than that of MIS-TLIF group at 2-year follow-up (12.3 ± 3.6% vs. 15.7 ± 4.8%, *P* < 0.001).

**Conclusion:**

Both PTES and MIS-TLIF show favorable clinical outcomes for LDD in elderly patients. Compared with MIS-TLIF, PTES has the advantages including less damage of paraspinal muscle and bone, less blood loss, faster recovery, lower complication rate, which can be performed under local anesthesia.

## 1. Introduction

With the progression of society, the improvement of living standards and the development of medical technology, the life span of human beings is continuously prolonged, and the number of patients with lumbar degenerative disease (LDD) is also increasing (1–3). LDD refers to a series of syndromes such as lumbago, leg pain or intermittent claudication caused by lumbar degeneration, mainly including lumbar disc herniation, lumbar spinal stenosis (lateral recess stenosis, intervertebral foramen stenosis, central spinal stenosis), degenerative spondylolisthesis of lumbar spine, degenerative scoliosis of lumbar spine, etc. Surgical treatment is needed for LDD that have poor effects of conservative treatment and seriously affect the quality of life. Conventional open surgery such as posterior lumbar interbody fusion (PLIF) or transforaminal lumbar interbody fusion (TLIF) has satisfactory clinical outcomes for LDD (4, 5). However, there are some disadvantages including extensive soft tissue dissection, large trauma, heavy bleeding, long postoperative recovery time, and high incidence of complications. Moreover, the patients with LDD are generally older, for whom the tolerance of open surgery is poor and the surgical risk is extremely high (6–9). How to reduce the surgical trauma of LDD has become very important. In recent years, minimally invasive surgery-transforaminal lumbar interbody fusion (MIS-TLIF) has been widely used in order to improve open TLIF, which can protect the attachment of paraspinal muscles to bone, avoid the disruption of supraspinous and interspinous ligaments, and decreased the approach-related complications (10).

With the deepening of minimally invasive concept and the improvement of surgical instruments, the clinical application of minimally invasive spine surgery (MISS) technology for the treatment of LDD is gradually being accepted, and spinal endoscopic surgery is the most minimally invasive technique (11, 12). In 2017, we first introduced the PTES (percutaneous transforaminal endoscopic surgery) technique with reduced steps, simple orientation and easy puncture, which can significantly decrease the times of fluoroscopy projection and shorten the operation time (13). It can effectively treat LDD with neurologic symptoms of lower extremities mainly caused by lumbar disc herniation, lateral recess stenosis, intervertebral foramen stenosis or central spinal canal stenosis. This study used PTES technique to treat LDD of elderly patients, which was compared with MIS-TLIF to evaluate their clinical efficacy, security and feasibility.

## 2. Materials and methods

### 2.1. Patients

84 elderly patients (>70 years old) of single-level LDD underwent the surgical treatment from November 2016 to December 2018. They were followed up for more than 2 years. 45 patients were treated with PTES in group 1, 39 patients treated with MIS-TLIF in group 2. The patients enrolling depended on the surgeon's experience and the patient's selection. The detailed data is shown in Table 1. This retrospective cohort study was approved by the medical ethical committee of Zhongshan Hospital Fudan University.

**Table 1:** Characteristics of the patients.

		PTES (45 cases)	MIS-TLIF (39 cases)
Age (years)		77.4 ± 5.9	76.0 ± 4.4
Gender (F/M)		20/25	18/21
LDD level (cases)	L3/4	7	5
	L4/5	26	22
	L5/S1	12	12
Calcification (cases)		7	5
Degenerative scoliosis (cases)		5	3
High iliac crest (cases)		4	2
Comorbidities (cases)	Hypertension	18	19
	Type II diabetes	7	5
	Anticoagulant therapy	6	2
	Coronary heart disease	3	4
	Cerebral infarction	3	3
	Hyperplasia of prostate gland	2	1
	Cataract	1	2
	Hepatitis	1	1
	History of humeral fracture	1	1
	Asthma	1	0
	Agitans paralysis	1	0
	History of rib fracture	1	0
	Nephritis or nephrotic syndrome	0	2
	Gout	0	1
	Myocardial infarction	0	1
	Rheumatoid arthritis	0	1
	Lung cancer	0	1
	Corticosteroid treatment	0	1
	History of tuberculosis	0	1
	History of appendectomy	5	6
	History of cholecystectomy	3	2
	History of hysterectomy	2	0
	History of cardiac ablation	2	0
	History of thyroid surgery	2	0
	History of gastrectomy	1	2
	History of Cesarean section	1	1
	History of total knee arthroplasty	1	1
	History of radical cystectomy	1	1
	Insertion of permanent pacemaker	1	1
	History of liver transplantation	1	0
	History of local mastectomy	1	0
	History of lienectomy	1	0
	History of colonic neostomy	1	0
	History of renal carcinomas surgery	1	0
	History of pelvic malignant tumor surgery	1	0
	History of lung surgery	1	0
	History of herniorrhaphy	0	2
	History of percutaneous vertebroplasty for lumbar fracture	0	1
Follow-up		44.9 ± 4.7	46.8 ± 6.3

Age and follow-up are expressed as the mean ± SD.

**Figure 1:**
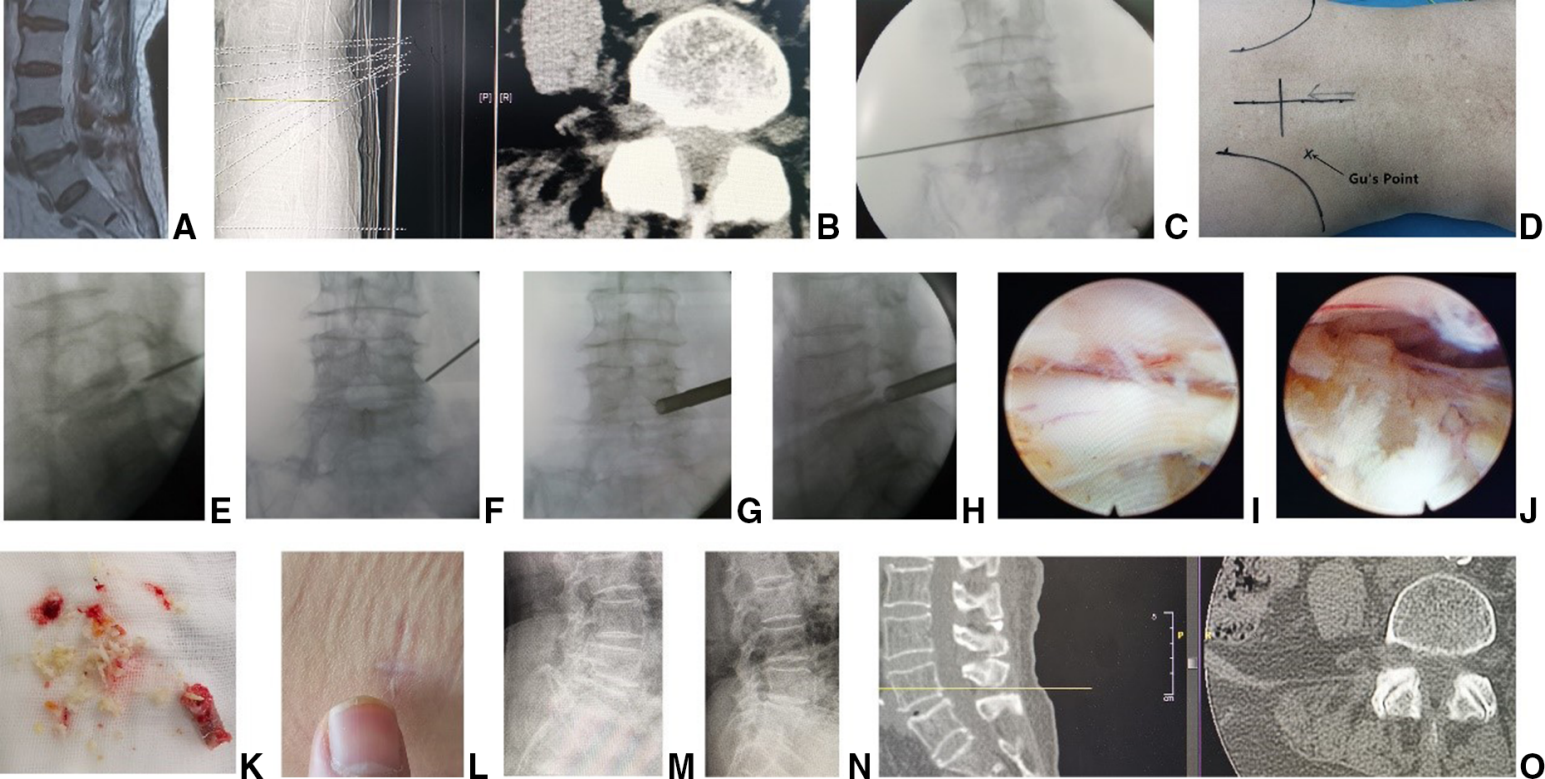
Female patient of 89 seats with LDD aL4 5 had bilateral asymmetric leg pain and the right side is more seven preoperative (**A**) sagittal MR and (**B**) CT images showed L4/5 disc herniation, lateral recess stenosis and neurologic compression. The procedure of PIES was undertaken for the patient. A transverse line bisecting the disc was draw along the metal rod which was placed transversely across the center of the target disc on (**C**) posteoanterior C-arm view. (**D**) Photography showed the surface marking of anatomic disc center identified by the intersection of transve: line and longitudinal midline, which was the aiming reference point of puncture, and the entrance point of puncture (Gu's point) located at the corner of flat back turning to lateral side. During puncture, once resistance disappeared, the C-arm view was taken to ensure that the tip of puncture needle was in the intracanal area clot to the posterior wall of disc on (**E**) lateral x-ray and near the lateral border of pedicle on (**F**) posteoanterior x-ray. During press down enlargement of foramen, when resistance disappeared, the tip of reamer should exceed the medial border of pedicle on (**G**) posteroanterior C-arm view and reach clot to the posterior wall of target disc on (**H**) lateral C-arm view. Under (**I,J**) endoscopic view, the bilateral compressed nerve roots were freed after (**K**) the hypertrophic ligamentum flavum and herniated disc were removed. (**L**) The picture showed the mini incision for PIES 2 years after surgery. No new lumbar instability was found on postoperative (**M**) hyperextension and (**N**) hyperflexion lateral x-ray image. (**O**) CT images showed the resected articular process and enlargement of L4/5 right foramen.

**Figure 2:**
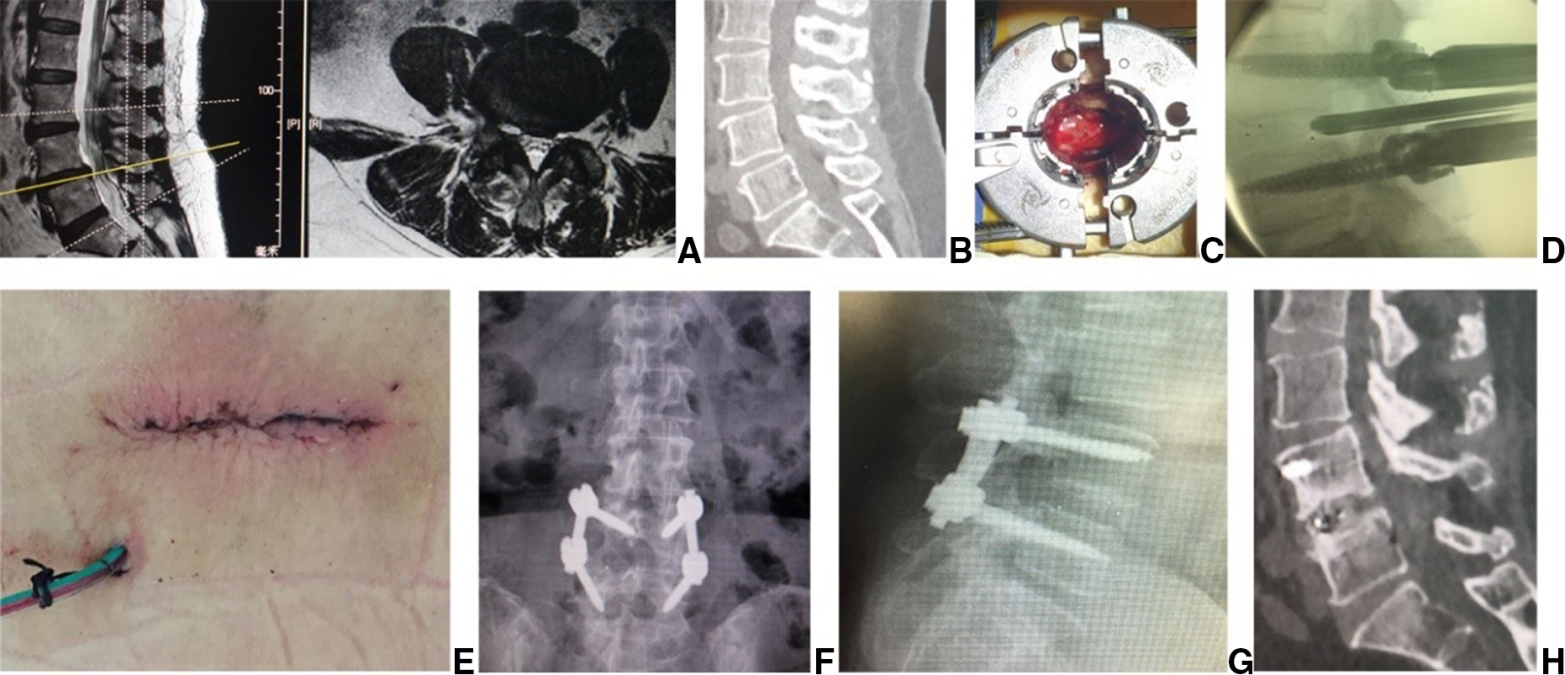
Female patient of 72 years with LDD at L4/5 had right leg pain. Preoperative (**A**) sagittal, axial MR and (**B**) CT images showed L4/5 disc herniation, lateral recess stenosis and neurologic compression. In MB-TLIF, (**C**) through an expandable tubular retractor, the dun and nerve root involved were exposed after a unilateral complete facetectomy and hemilaminectomy. (**D**) The fluoroscopic image showed that a cage filled with autograft bone was inserted after the disc material and cartilaginous endplate were removed. Two rods were fixed over pedicle screws and (**E**) the wound was closed in layers with the drainage tube placed. (**F,G**) Postoperative x-ray confirmed that the position of pedicle screws and cage was good. Fusion grade based on the Bridwell grading system at 2 year follow-up WU Grade I on (**H**) sagittal CT.

Inclusion criteria: (1) the clinical symptoms were unilateral leg pain, bilateral legs pain or intermittent claudication. (2) Image data such as MRI and CT showed a single-level LDD of lumbar disc herniation, intervertebral foramen stenosis, lateral recess stenosis or central spinal stenosis from L1 to S1, which was consistent with the clinical symptoms of corresponding neurologic compression (Figures 1A,B, 2A,B). (3) The outcome after at least 3 months of regular conservative treatment was poor. (4) The patient's age was over 70 years old, the systemic status was good with independent understanding and thinking ability, basic medical diseases were under control, (5) the patient can be followed up for at least 2 years.

Exclusion criteria: (1) imaging examination showed lumbar spondylolisthesis or intervertebral instability. (2) Lumbar spine inflammation, tumors and other lesions. (3) Coagulation dysfunction, infection in the surgical area.

### 2.2. Pre- and postoperative imaging

Peoperative MRI and CT imaging (Figures 1A,B) were used to determine the involved segment and to determine if there was calcification. Posteroanterior and lateral radiographs were obtained to detect spondylolisthesis, scoliosis or high iliac crest when the lower plate of L4 vertebral body was not higher than the line between highest points of bilateral iliac crest. Postoperative MRI images were used to assess neurologic decompression or exclude reherniation. Postoperative CT was obtained to assess the facet joint after press-down enlargement of foramen in PTES, and the position of pedicle screws and cage in MIS-TLIF. The fusion status was assessed on CT according to the Bridwell's fusion grades (14). The hyperextension and hyperflexion lateral x-ray were used to check intervertebral instability after surgery.

### 2.3. Surgical procedure

C-arm was used for intraoperative fluoroscopic imaging. Operative duration (PTES/from patient's position to closure of incision, MIS-TLIF/from cut to closure of incision), blood loss, intraoperative fluoroscopy frequency, incision length, and hospital stay were recorded for subsequent analysis.

#### 2.3.1. Group 1: PTES

**Figure 3:**
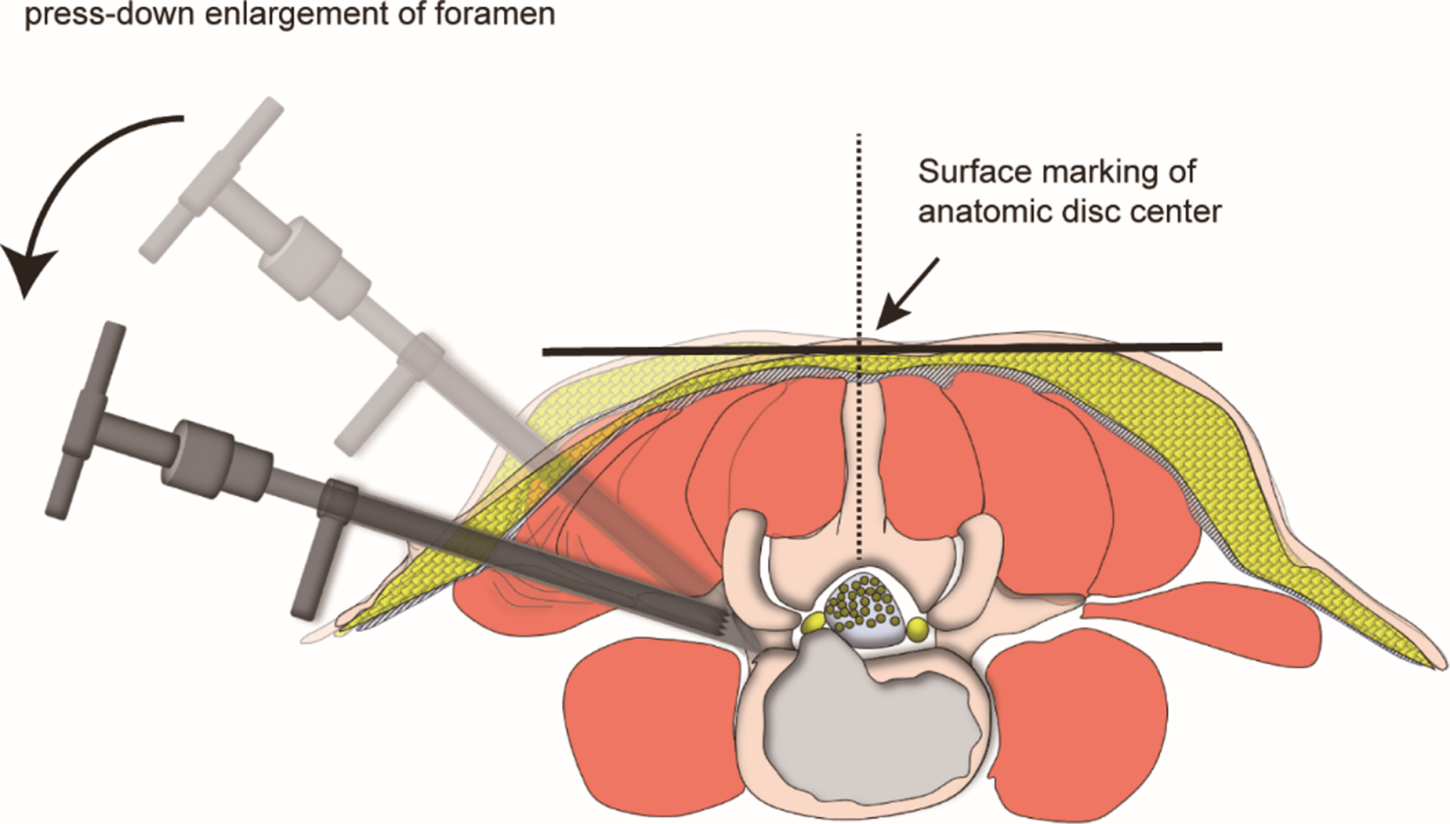
Schematic diagram of “press-down enlargement of foramen” in FITS. The 8.8-mm protective cannula was docked at the facet joint and pressed down further to make the angle of cannula to horizontal plane smaller, and a 7.5-mm trephine was introduced to cut the ventral bone of articular process for the enlargement of intervertebral foramen, which made the working channel easily inserted into the spinal canal even if the puncaire angle was 85° to the horizontal plan.

PTES was performed under local anesthesia with 1% lidocaine combined with appropriate intravenous analgesics. The patient was placed in a prone position with soft bolsters under the abdomen on a radiolucent table, making the hip joint flexion and keeping the back in a horizontal state. On the skin of back, draw the median line according to lumbar spine process, and the location line of surgical level determined by posteroanterior C-arm fluoroscopy (Figure 1C). The intersection of location line and midline was the surface projection of anatomical center of intervertebral disc. The puncture point was located at the corner of flat back turning to lateral side according to “Gu's point” (13, 15) (Figure 1D). The puncture needle was inserted at 25°- 85° to the horizontal plane aiming at the vertical line of body surface through the anatomical center of intervertebral disc. After the successful puncture, the needle should reach the posterior 1/3 of intervertebral space or around its posterior edge on the lateral C-arm fluoroscopy (Figure 1E), and near the outer edge of pedicle on the posteoanterior film (Figure 1F). The guiding wire was inserted through the puncture needle and a nearly 8 mm small incision was made. After stepwise dilation, the thick guiding rod of 6.3 mm in diameter was introduced over the guiding wire into the foramen. Then over the guiding rod, the 8.8-mm protective cannula was inserted, and docked at the facet and pressed down to make the angle of cannula to the horizontal plane smaller according to the inclination of puncture needle on the fluoroscopy. A 7.5 mm reamer was introduced to cut the outer and ventral bone of articular process, which was press-down enlargement of foramen (13, 15) (Figure 3). After resistant disappearing, the fluoroscopy showed that the top of reamer exceeded the inner edge of pedicle on the posteoanterior film (Figure 1G) and reached the posterior edge of target intervertebral space on the lateral film (Figure 1H). For lumbar central spinal canal stenosis, press-down enlargement of foramen was repeated to remove more bone of ventral part of articular process for enlargement of central spinal canal. A 7.5 mm working channel was placed along the guiding rod and the ipsilateral traversing and exiting nerve root, contralateral traversing nerve root, or epidural sac were exposed under direct vision (Figures 1I,J) after the hypertrophic ligamentum flavum and protruding disc tissue (Figure 1K) were removed for the open of lateral recess and central spinal canal. Sometimes the central and contralateral posterior longitudinal ligament, annulus fibrosus or nucleus pulposus need to be removed using flexible bipolar radiofrequency and angled nucleus pulposus forceps for clearer exposure of contralateral nerve root.

After the procedure, the patients rested in bed except for going to toilet for 3 days. The functional exercise began 3 days after surgery, and they can go to work 1 week after surgery. External braces were used for 2 weeks.

#### 2.3.2. Group 2: MIS-TLIF

The patient was placed in the prone position on a radiolucent operating table under general anesthesia. After orientation with fluoroscopy, the superior facet joints and transverse processes of upper and lower vertebrae were exposed through a 3.5 cm midline incision and bilateral paraspinal muscle-splitting approaches. The pedicle screws (DePuy, Inc., Warsaw, IN, United States) were inserted into the vertebrae on the junction between the lateral border of superior articular facet and midline of transverse process. Then, an expandable tubular retractor (DePuy, Inc., Warsaw, IN, United States) was placed along the stepwise dilation. The dura and nerve root involved were exposed after a unilateral complete facetectomy and hemilaminectomy (Figure 2C), and the disc material and cartilaginous endplate were almost totally removed. The autologous bone graft was packed into the disc space, and a cage (DePuy, Inc., Warsaw, IN, United States) filled with autograft bone was obliquely inserted (Figure 2D). Two rods were fixed over pedicle screws, the suction drain was placed and the wound was closed in layers (Figure 2E).

The drainage tube was removed when drainage volume was less than 20 ml/24 h, then the patients were mobilized as soon as feasible and encouraged to resume their daily routine after leaving the hospital. External braces were used for 3 months after the procedure.

### 2.4. Clinical follow-up

The patients were followed-up in the outpatient. Visual analogue scale (VAS) was used to score the pain of low back and lower limbs before and immediately after surgery, 1 month, 2 months, 3 months, 6 months, 1 year and 2 years. Oswestry disability index (ODI) was collected before and 2 years after surgery. All complications such as iatrogenic nerve injury, infection, myocardial infarction, cerebral infarction and recurrence were documented during the follow-up.

### 2.5. Statistical analysis

SPSS 25 software (SPSS Inc., Chicago, IL, United States) was used to perform statistical analysis, and a value of less than 0.05 was considered statistical significance. Normal distributed continuous variables such as age, operative duration, incision length, follow-up and ODI are presented as mean ± standard deviation (SD); Discrete, rating variables and continuous variables, which are not normally distributed, are presented as median (Maximum- Minimum) including fluoroscopy frequency, blood loss, drainage removal, hospital stay, VAS; Categorical variables such as gender, basic diseases, lumbar level, rates of LDD with calcification, scoliosis and L5/S1 LDD with high iliac crest are expressed as frequency or percentage. Student t-test is used for intergroup analysis of normal distributed continuous variables. The Mann–Whitney *U*-test is used for intergroup analysis of discrete variables, rating variables and continuous variables, which are not normally distributed. The chi-square test is used for intergroup analysis of categorical variables. The Kruskal–Wallis test followed by the Dunn procedure with Bonferroni correction is performed for intragroup comparison of VAS at different time points. The ODI score before surgery and 2 years after surgery are compared using student *t*-test.

## 3. Results

Clinical data are summarized in Table 1. There are no significant differences in age, gender, basic diseases, lumbar level and follow up between two groups. PTES group has 7 patients of LDD with calcification, 5 with lumbar degenerative scoliosis, 4 with high iliac crest (L5/S1) and MIS-TLIF group has 5 patients of LDD with calcification, 3 with lumbar degenerative scoliosis, 2 with high iliac crest (L5/S1). There are significantly less operative duration (55.6 ± 9.7 min vs. 97.2 ± 14.3 min, *P* < 0.001), less blood loss [11(2–32) ml vs. 70(35–300) ml, *P* < 0.001] (Figures 1L, 2), shorter incision length (8.4 ± 1.4 mm vs. 40.6 ± 2.7 mm, *P* < 0.001), less fluoroscopy frequency [5(5–10) times vs. 7(6–11) times, *P* < 0.001] and shorter hospital stay [3(2–4) days vs. 7(5–18) days, *P* < 0.001] in PTES group than those in MIS-TLIF group. The drainage tube was removed 4(3–7) days after surgery in MIS-TLIF group. (Table 2).

**Table 2:** Operation-related data in PTES and MIS-TLIF.

	PTES (45 cases)	MIS-TLIF (39 cases)
Operative duration(minutes)	55.6 ± 9.7	97.2 ± 14.3*
Blood loss (ml)	11 (2–32)	70 (35–300)*
Incision length (mm)	8.4 ± 1.4	40.6 ± 2.7*
fluoroscopy frequency (times)	5 (5–10)	7 (6–11)*
Removal of drainage tube (days)	/	4 (3–7)
hospital stay (days)	3 (2–4)	7 (5–18)*

Operative duration and incision length is expressed as the mean ± SD. Blood loss, fluoroscopy frequency, removal of drainage tube and hospital stay is expressed as the median (minimum–maximum).

* *P* < 0.001, significant difference between two groups.

The back VAS significantly decreased from 7(4–10) before surgery to 0(0–1) after surgery in PTES group (*P* < 0.001), while preoperative VAS scores of back pain was 6(4–10), which dropped to 3(2–5) immediately after surgery, 1(0–2) at 1-month and at 2-year follow-up in MIS-TLIF group (*P* < 0.001). The preoperative leg VAS scores significantly decreased after surgery in both groups (*P* < 0.001), and there was no statistical difference of leg VAS scores after surgery between two groups. However, back VAS scores in PTES group were significantly lower than those in MIS-TLIF group at any time point after surgery (*P* < 0.001). The preoperative ODI was 67.8 ± 9.1% and 68.7 ± 9.5% in PTES group and MIS-TLIF group, respectively. It significantly dropped in both groups (*P* < 0.001) but the ODI of PTES group was significantly lower than that of MIS-TLIF group at 2-year follow-up (12.3 ± 3.6% vs. 15.7 ± 4.8%, *P* < 0.001) (Table 3).

**Table 3:** VAS and ODI before and after operation.

		Pre-op	Post-op	1 month	2 months	3 months	6 months	1 year	2 years
Back pain VAS	PTES	7 (4–10)	0 (0–2)*^,#^	0 (0–1)*^,#^	0 (0–1)*^,#^	0 (0–1)*^,#^	0 (0–1)*^,#^	0 (0–1)*^,#^	0 (0–1)*^,#^
	MIS–TLIF	6 (4–10)	3 (2–5)^#^	1 (0–2)^#^	1 (0–2)^#^	1 (0–2)^#^	1 (0–2)^#^	1 (0–2)^#^	1 (0–2)^#^
Leg pain VAS	PTES	8 (6–10)	1 (0–3)^#^	0 (0–2)^#^	0 (0–2)^#^	0 (0–2)^#^	0 (0–2)^#^	0 (0–2)^#^	0 (0–2)^#^
	MIS–TLIF	8 (7–10)	1 (0–3)^#^	0 (0–2)^#^	0 (0–2)^#^	0 (0–2)^#^	0 (0–2)^#^	0 (0–2)^#^	0 (0–2)^#^
		Pre-op				2 years			
ODI (%)	PTES	67.8 ± 9.1				12.3 ± 3.6*^,#^			
	MIS–TLIF	68.7 ± 9.5				15.7 ± 4.8^#^			

VAS is expressed as the median (minimum–maximum). ODI is expressed as the mean ± SD.

* *P* < 0.001, significant difference between two groups at the same time point after surgery.

^#^ *P* < 0.001, significant difference between preoperatively and postoperatively in the same group.

There was no new intervertebral instability after PTES on lumbar hyperextension and hyperflexion lateral radiographs (Figures 1M,N) although the facet joint was involved (Figure 1O) in some cases, and no recurrence was found in PTES group. No complications of wound infection, permanent nerve injury and rupture of large vessels occurred. However, 2 patients had cardiac infarction about 2 days after surgery and were cured after thrombolytic therapy in MIS-TLIF group. There was no hardware failure (Figures 2F,G), and fusion grades based on the Bridwell grading system at 2-year follow-up were grade I (Figure 2H) in 24 segments (61.5%, 24/39), grade II in 15 segments (38.5%, 15/39). One patient developed LDD at adjacent segment 4 years after surgery and received PTES with good outcomes in MIS-TLIF group.

## 4. Discussion

In China, the fastest growing population is projected to be persons >70 years old. LDD are associated with advancing age, and the number of patients experiencing LDD has significantly increased (16, 17). Patients with LDD typically present with pain, functional limitations, and neurologic deficits, and they often demand interventions that will improve their quality of life. However, in these aged patients who often harbor multiple medical comorbidities, such open surgeries of neurologic decompression and intervertebral fusion pose a technical challenge associated with a high risk of intraoperative and postoperative complications (6–9). Since Foley et al. introduced the MIS-TLIF procedure to reduce the approach related muscle damage (18, 19), many investigators have reported significant advantages on open PLIF and TLIF. Its advantages include, but are not limited to, less intraoperative blood loss, less postoperative pain, decreased postoperative narcotic usage, early ambulation, and decreased length of hospital stay (18–24). However, MIS-TLIF should be performed under general anesthesia, which needs sedative medication, muscular relaxant, endotracheal intubation and ventilator, and has comprehensive and adverse impact on the respiratory system, circulatory system, nervous system, urinary system and so on. These make the elderly patients of LDD undergoing MIS-TLIF in great danger. Additionally, the senile osteoporosis decreases the pull-out resistance of pedicle screws and increases the rate of hardware failure. Whereas, PTES under local anesthesia can avoid these shortcomings (13, 15), and PTES is of cost saving because local anesthesia is much cheaper than general anesthesia. The results of this study show that PTES has significantly less blood loss, shorter incision length and shorter hospital stay compared with MIS-TLIF. The cardiac infarction occurred in 2 patients after MIS-TLIF, and there was no complication in PTES group.

Most patients with LDD have the symptoms of nerve root compression, such as one leg pain, or bilateral symmetric or asymmetric legs pain. Some patients have intermittent claudication of lower limbs with no neurologic symptoms when rest and have pain, numbness, discomfort or tiredness of single or bilateral lower limbs after walking for 50 to 100 meters, which could be relieved after a few minutes of rest. We use PTES technique to treat LDD with neurologic symptoms. During the procedure, we perform press-down enlargement of foramen to saw off the ventral bone of articular process (13, 15) (Figure 3), and the hypertrophic ligamentum flavum and the protruding nucleus pulposus are removed to expand the lateral recess and reduce the pressure of nerve roots. When the patients of LDD has central spinal canal stenosis, press-down enlargement of foramen is repeated to remove more ventral bone of articular process and expose dural sac, ipsilateral and contralateral nerve roots (Figures 1I,J) for almost 180° enlargement of central spinal canal. The bilateral nerve roots can be decompressed from unilateral side through a small incision (Figure 1L). The results of this study show that preoperative leg pain of neurologic symptoms was significantly relieved after PTES, which was same as group 2 of MIS-TLIF, and there was no statistical difference of leg VAS scores after surgery between two groups. The preoperative ODI significantly dropped at 2-year follow-up in both PTES and MIS-TLIF group. These are in accordance with those studies of comparing endoscopic surgery with MIS-TLIF for LDD (25).

The orientation of PTES is simple, and it is only needed to take the posteroanterior fluoroscopy to determine the horizontal location line of surgical level (Figure 1C) The target of puncture is the vertical line through the intersection of location line and lumbar back midline (Figures 1D, 3) The entrance point of puncture is located at the corner of flat back turning to lateral side, which does not depend on age, gender, body size and fluoroscopic image. We named it “Gu's point” (13, 15) (Figure 1D), which is closer to the midline than that in other posterolateral endoscopic surgery, and there are four advantages: (1) Avoid injuring the exiting nerve root. Exiting nerve root leaves the foraminal in the direction from superomedial to inferolateral. If the entrance point locates laterally, the foraminotomy procedure may meet and injure the exiting nerve root more possibly and the patient may complain of pain in lower extremities during surgery. (2) Avoid blockage by the high iliac crest for the L5/S1 level. Peak of the iliac crest locates at the lateral side of waist and the height lowers down when getting closer to the midline. Height of iliac crest at “Gu”s Point” is relatively lower, reducing the difficulty of puncture and subsequent operation for L5/S1. (3) Shorten the surgical path. The entrance point is more lateral from the midline, which makes the path for surgical target longer. Especially in obesity patients, more subcutaneous adipose tissue makes the puncture point more distal from surgical target, which needs very long working channel for transforaminal endoscopic surgery. (4) Avoid injuring abdominal viscera and main blood vessels. Puncture from a lateral entrance point is easy to penetrate into the abdomen. Puncture from “Gu”s Point” is much safer, the tip of needle could be blocked by the bony structure of spine even if in a large angle of inclination. Once the needle gets the target, it is acceptable that the tip of needle is in the posterior one third of disc on the lateral x-ray, which makes the puncture of PTES easy. “Press-down enlargement of foramen” (13, 15) (Figure 3) can let reamer remove the ventral bone of articular process and make it easy to place the working cannula into spinal canal between dural sac and disc, although the puncture needle is in intervertebral space. Instead of stepwise manipulation, the one-step enlargement of foramen is performed using 7.5 mm reamer during PTES. This reduced steps, simple orientation and easy puncture can significantly decrease the frequency of fluoroscopy projection and shorten the operation time. This study shows that the fluoroscopy frequency is 5 (5–10) times and the operation time is 55.6 ± 9.7 min from body position to incision closure in PTES group, which are significantly less than those in MIS-TLIF group.

In this study, although press-down enlargement of foramen involved the facet joint in some cases, no new intervertebral instability was observed after PTES on lumbar hyperextension and hyperflexion lateral radiographs and internal fixation was not needed. There was no recurrence in PTES group, which is closely related to strict lumbar care after surgery. The protruded nucleus pulposus is removed under endoscope, and the remaining portion in the intervertebral space is healthy and relatively intact, which can keep stable and will not protrude again. If neglecting postoperative waist maintenance, the remaining nucleus pulposus may rupture and protrude again. It is very important to repeatedly remind the patients not to bend down, not to lift heavy objects, not to maintain a same posture for a long time, and not to focus strength on the waist when coughing and sneezing, which can effectively prevent LDD recurrence. In MIS-TLIF group, LDD at adjacent segment occurred 4 years after surgery in one patient, which also correlates with bad postoperative care and concentration of stress on the adjacent segment. This adjacent segment disease can still be treated with PTES under local anesthesia.

There are also some limitations in this study. It is a single-center retrospective study with a relatively small number of patients. There is no comparison of PTES with other spinal endoscopic techniques. Therefore, we will perform a multicenter prospective controlled study to compare PTES with Yeung Endoscopic Spine Surgery (YESS), Thomas Hoogland Endoscopic Spine Surgery (TESS), Full Endoscopic Discectomy (FED), Unilateral Biportal Endoscopy (UBE) for the treatment of LDD.

In conclusion, both PTES and MIS-TLIF show favorable clinical outcomes for LDD in elderly patients. Compared with MIS-TLIF, PTES has the advantages including less damage of paraspinal muscle and bone, less blood loss, faster recovery, lower complication rate, and can be conducted under local anesthesia.

## Data Availability

The raw data supporting the conclusions of this article will be made available by the authors, without undue reservation.
